# Trainings in Health Sector: Need for a Paradigm Shift

**DOI:** 10.4103/0970-0218.58379

**Published:** 2009-10

**Authors:** Shiv Chandra Mathur

**Affiliations:** Department of Preventive and Social Medicine, SMS Medical College, Jaipur 302004, India. E-mail: shiv_mathur@hotmail.com

*‘Sometimes we, a nation of a billion people, think like a nation of a million people.’*
**P. J. Abdul Kalam**

India stagnant at bottom level on the HDI scale year-after-year is a matter of concern.(1) High morbidity and mortality rate substantially contributes to this undesired phenomenon. Although continuing gap in the health status of the people may be attributed to several factors, but it can be said with certainty, that health systems performance is seriously affected by poor development of human resources for health.(2,3)

Within this decade, there has been a phenomenal increase in human resources in the health sector, yet the health systems are far from achieving the desired status. Poor outcome indicators reflect the need for high level of concern, commitment and competence among the health personnel responsible for the delivery and management of health care, particularly at the primary care facilities. Continuous efforts have been made to address these issues by providing a variety of trainings to health care providers. At this crucial juncture, there is a need to re-examine the status of training and the strategic changes required in this context.

Most of the in-service trainings in the health departments of different states are either part of centrally sponsored schemes or projects funded through Government of India. Sequel of this approach is manifested in terms of several trainings often occurring concurrently for the same level of professionals. More often, the trainees in these programs are nominated randomly in compliance to orders reaching to the nominating authorities at eleventh hour. Trainees have to reach to the training venue at a very short notice. Consequently trainings - which are loaded with lectures with little respect for continuum of the theme and overall perspective of the program - are compromised with low number of trainees. Provision of per diem for trainees and remuneration for trainers also fluctuates from program to program and influence the attendance and *spirit* of participation. Profiles of the trainees often reflect the mismatch between organization goals and their personal goals. Eventually, the subjects nominated for training and watching them post-training on work-site often do not give the signals of capacity development of the concerned health systems. These are well identified challenges which have remained unmet for decades.(4)

Professional Development Courses (PDC) of ten weeks duration for district level Medical Officers were initiated as a part of health sector reforms in the beginning of this decade. Starting from National Institute of Health and Family Welfare, these were taken down to fourteen institutions throughout the country and more then 900 doctors were oriented in PDCs over a period of first five years of its launch. In most of these programs, the profile of the participants nominated remained a matter of debate. Subsequent use of trained doctors and their contribution to the respective State Health Departments remains a matter of debate.

Trainings envisaged in each of the health sector project have several common components addressed to the same professional i.e. training on RTI/STI in RCH as well as NACP-3. It is also not easy to cope up with several trainings envisaged in the same project without having a lucid PMIS with the authorities nominating the trainees. RCH-2, (which is a now a major activity within the National Rural Health Mission) has trainings on skill building for Emergency Obstetrical Care (EmOC), Medical Termination of Pregnancy (MTP), Intra Uterine Device (IUD) insertion, laparoscopy, anesthesia for EmOC, Adolescent Friendly Health Services (AFHS), Integrated Management of Childhood Illnesses (IMNCI), Non Scalpel Vasectomy (NSV), Behavior Change Communication (BCC), Routine Immunization and Emergency Contraception. Pace with which the changes in the health systems occur after these trainings places back questions on the quality and methods of trainings. Take for example, AFHS trainings suggested by Government of India in its RCH guidelines to address the reproductive health issues in the adolescence. In Rajasthan, by the fourth year of RCH-2, Senior Medical Officers from 67 Community Health Centers (CHCs) were oriented at State level but none of the CHC has yet established full time Adolescent Clinic in their facility. Similarly, procurement of mannequins for Medical Colleges took one-and-half year after the faculty of Anesthesia was oriented on strategy for building skills of Medical Officers working at rural health facilities for providing anesthesia in emergency obstetric care. Contribution of Doctors trained in anesthesia over last three years in strengthening EmOC services has become a matter of investigation! Such instances can be observed in many other states. Thus lack of coordination between collateral activities and training defeats the very purpose for which training is imparted.

Clinical skill development trainings envisaged in the Health System Development Projects (HSDP) had a similar fate. It took three to four years for each State to identify an institute, delegate the training and by the time trainings were initiated, it was time to wind up HSDP! Review Missions of HSDPs from World Bank have observed same phenomenon in State after State, be it Karnataka, Uttaranchal or Rajasthan. One of the major reasons for this weakness is embedded in poor dialogue between health service delivery system which caters to primary and secondary care on one hand, and medical education departments addressing tertiary level care on the other hand. Weak linkage between the two was at the core of ineffectiveness of the skill based trainings conducted.

It seems that health systems also do not want to give up their illusion that all trainings can be imparted through State Institutes of Health and Family Welfare (SIHFW) and Health and Family Welfare Training Centers (HFWTC). These institutions – if managed properly – can organize management trainings of quality but they neither possess the clinical resources, nor the capacity to interact with clinicians to drive skill based training to the extent desired by the delivery system.

Project based training approach over the years has also generated a culture where implementers have started demanding training for every small activity. Health systems seem to work on the premise that whether it is Auxiliary Nurse Midwife (ANM), Staff Nurse or Medical Officer, each of them need trainings even on their basic functions. What are then ANM Training School, General Nursing and Midwifery School or for that matter a Medical College is doing? Other side of the coin demands introspection about imparting skills in basic curricula of each of these courses. How many students ever attempt to inculcate skills for intra-dermal injection or IUD insertion? Why Government in each State is investing in intern's training when everything has to be taken anew in the in-service training?

Health Systems of the States have also yet to evolve a firm strategy for induction training which is well set for almost all other civil services i.e. general administration, police, accounts, forest etc. Wherever and whenever induction trainings have been tried by the health departments, they have neither been in consonance with induction, nor were they attempted rationally on need basis. Such an approach has added to the cost of in-service trainings to be imparted subsequently.

This is a high point when ambivalence, confusion and differences in points of view on various facets of training need to be curbed. In this background, each State in the country needs a health system specific training policy, a human resource policy, a personnel management system and above all commitment to implement each policy *in spirit*. Concurrently training cadre has to be strengthened by grooming the trainers of SIHFW and HFWTC in a meticulous manner.(5)

Training should be seen as a part of the overall process of human development. There is an urgent need to change the current training paradigm in health sector from knowledge and competence building to organizational transformation. The time has come to seriously consider training as an intervention. While training should emphasize skills development to perform tasks effectively, training designs should be reoriented to ensure a change in the attitude and mindset of health care providers at all levels to achieve high organizational and professional commitment.(6) Faculty of Preventive and Social Medicine can play an instrumental role in making health training systems effective.
